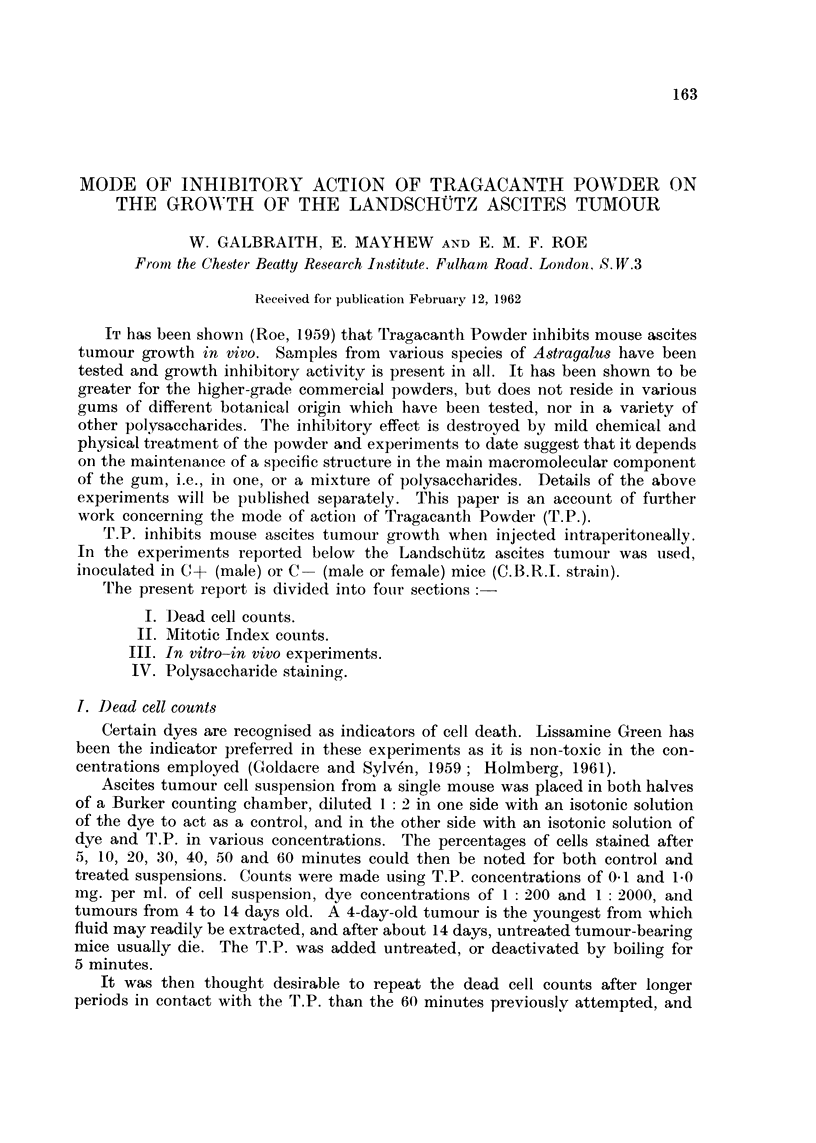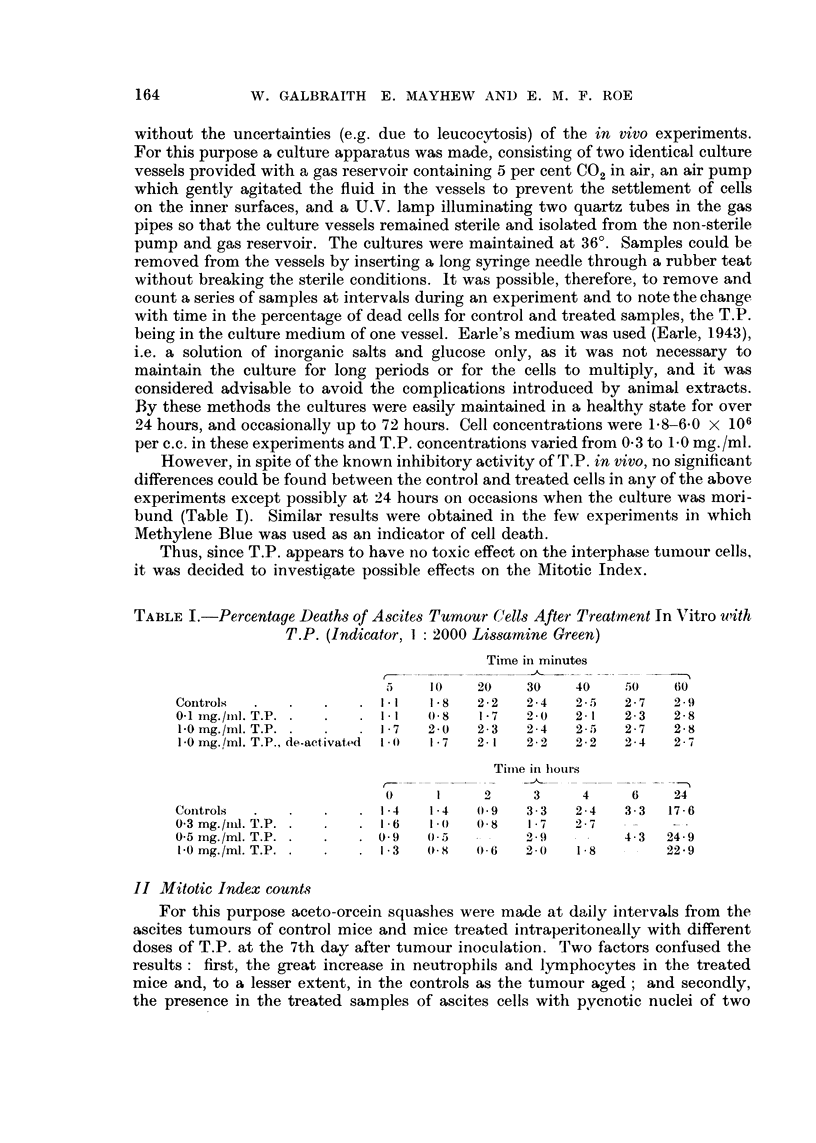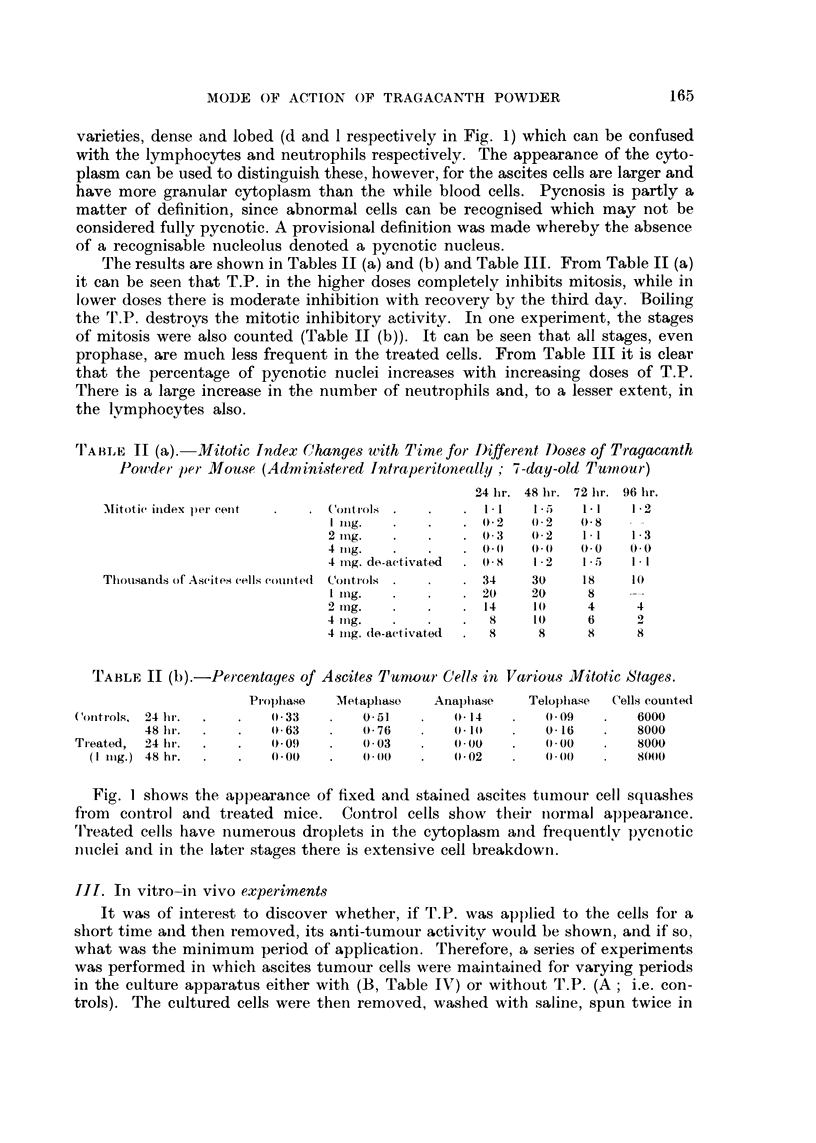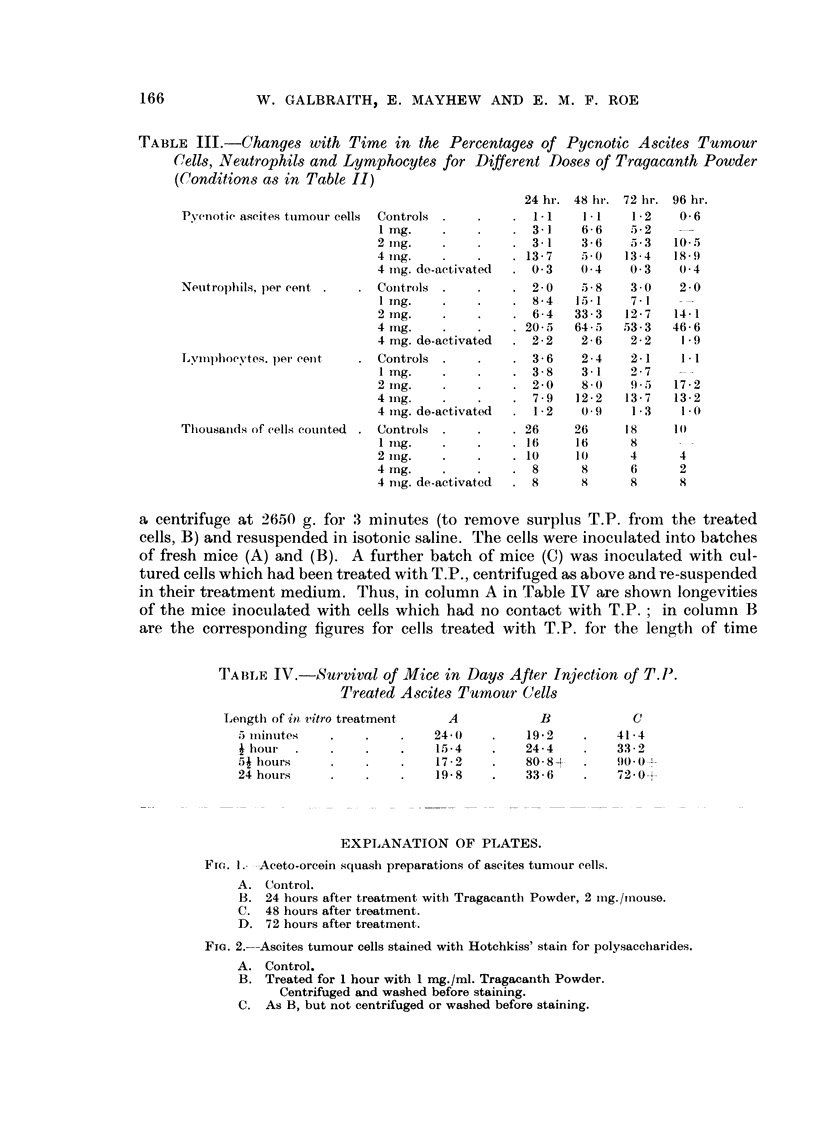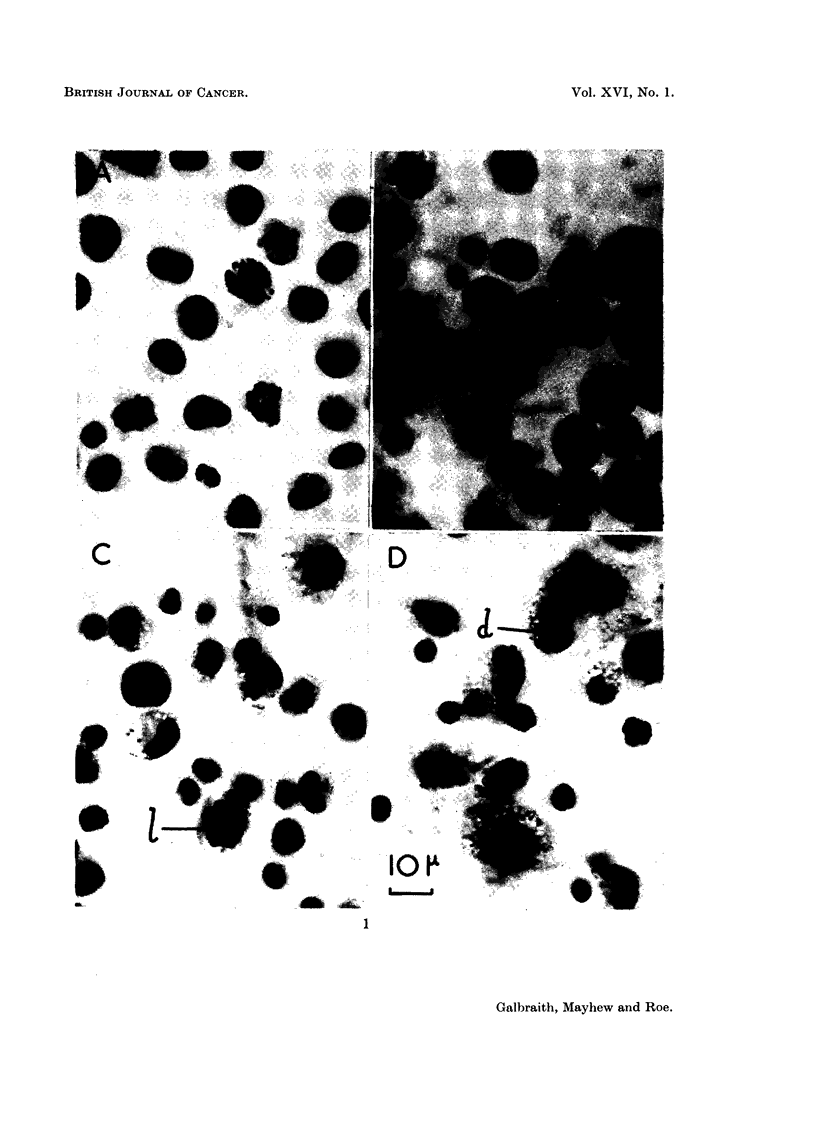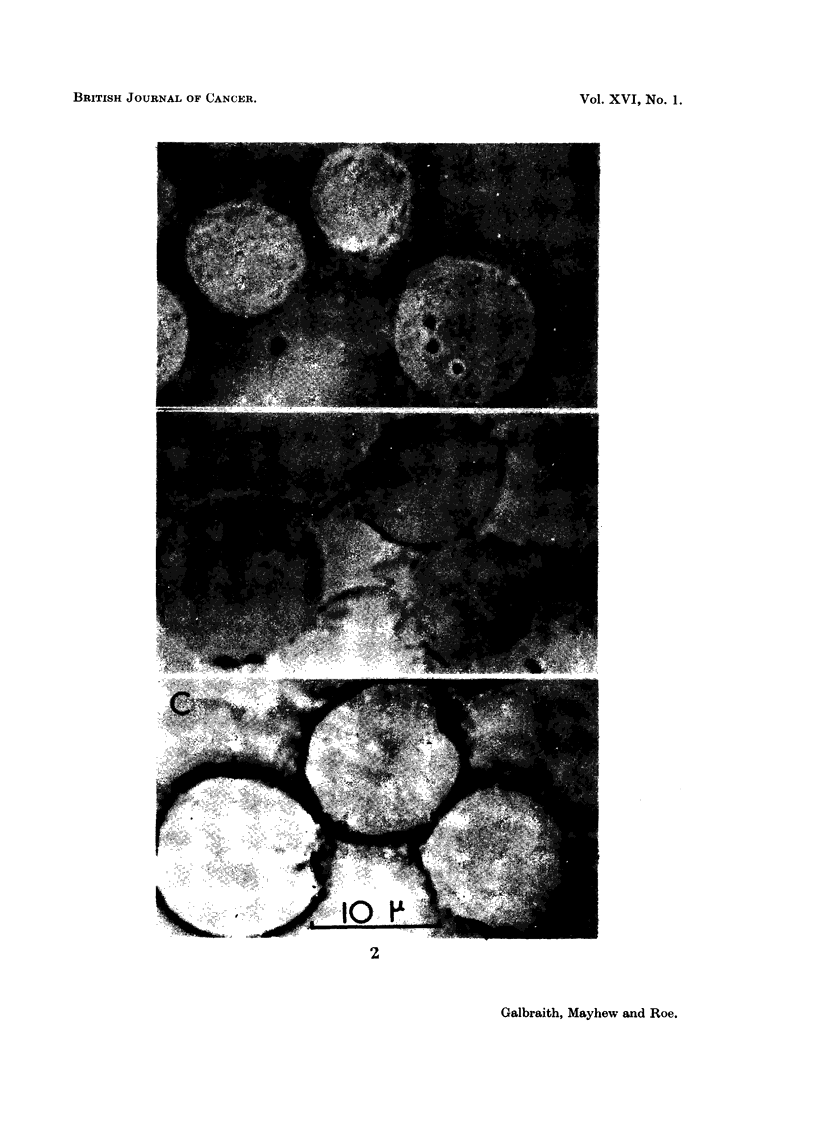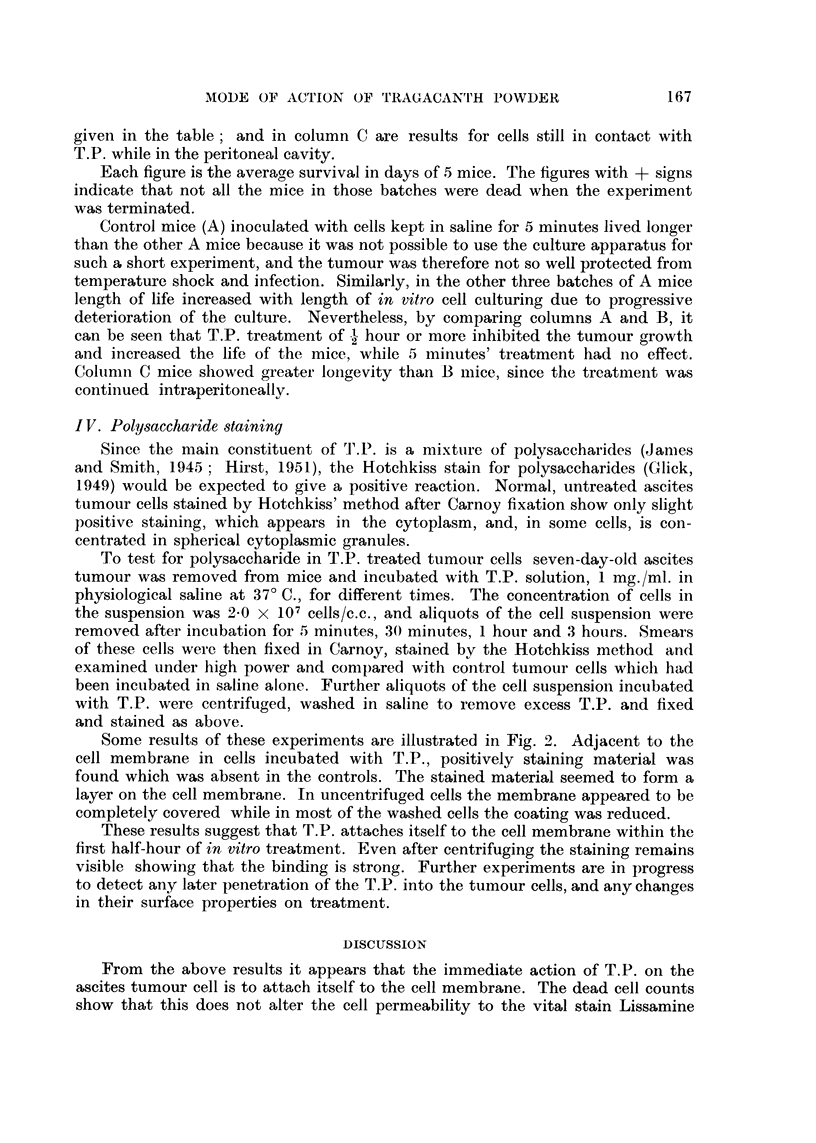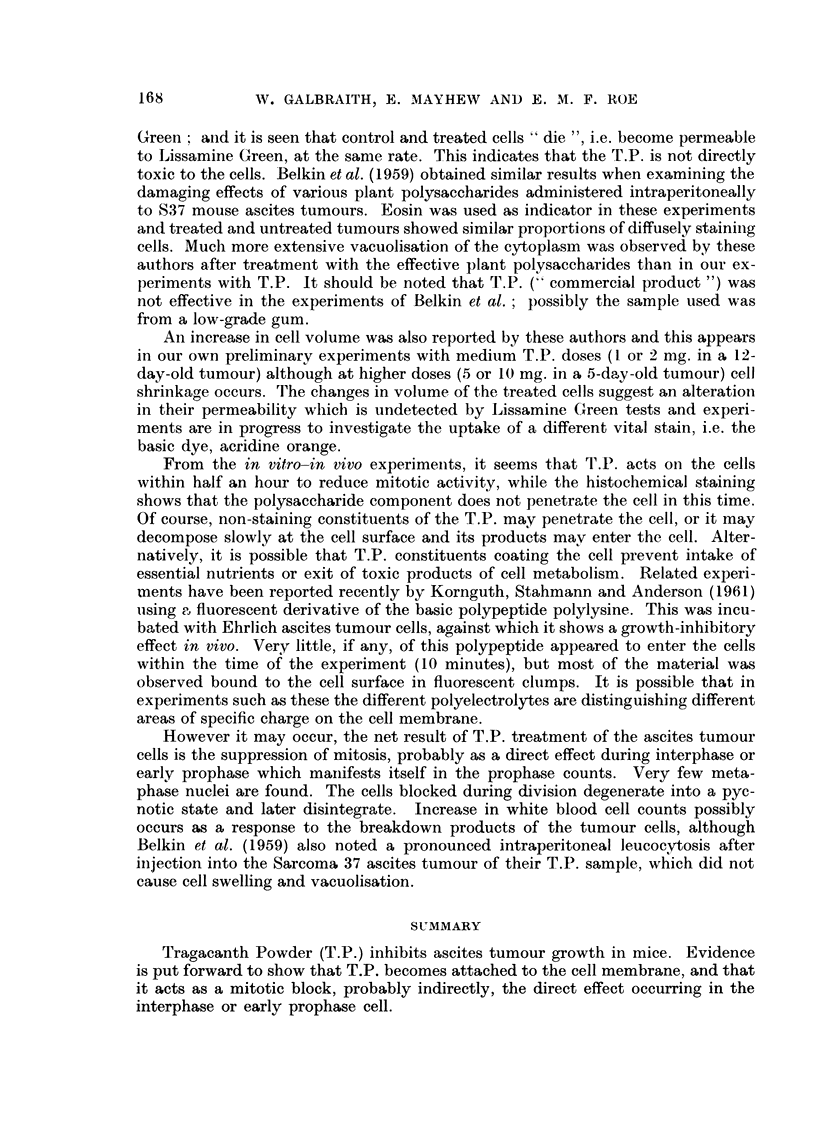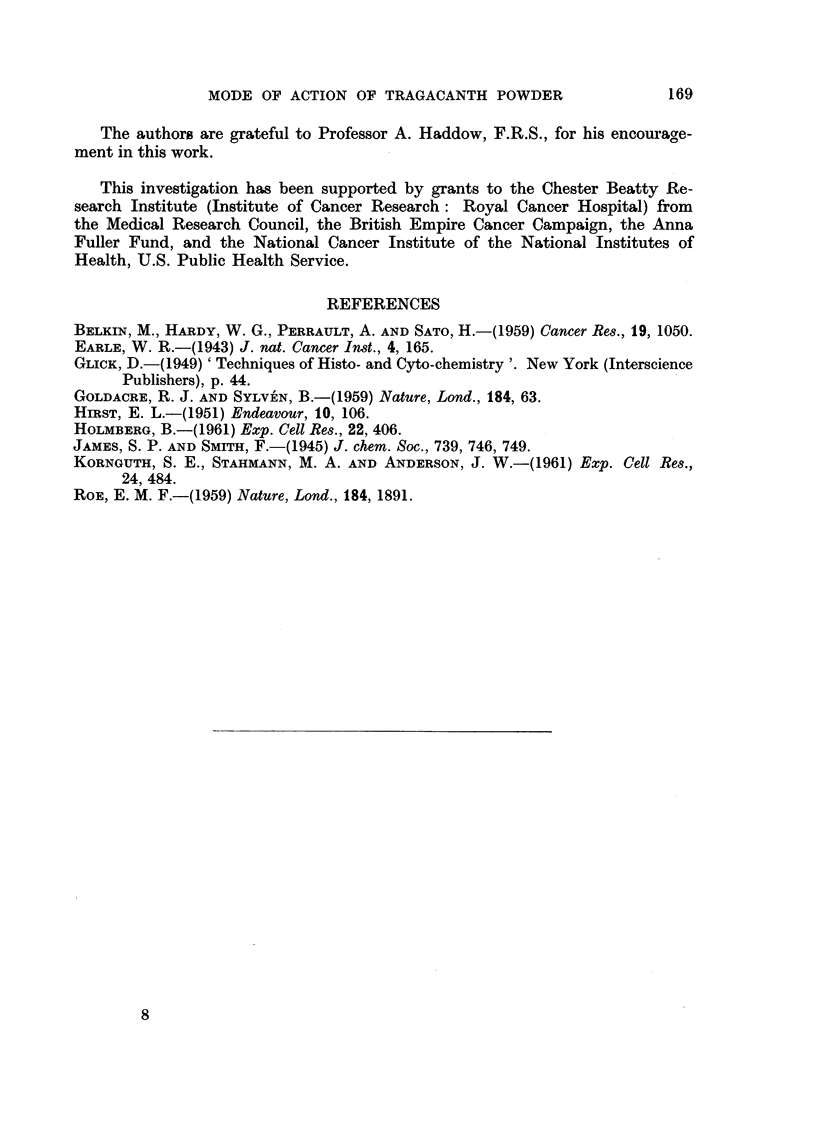# Mode of Inhibitory Action of Tragacanth Powder on the Growth of the Landschütz Ascites Tumour

**DOI:** 10.1038/bjc.1962.17

**Published:** 1962-03

**Authors:** W. Galbraith, E. Mayhew, E. M. F. Roe

## Abstract

**Images:**


### MODE OF INHIBITORY ACTION OF TRAGACANTH POWDER ON THE GROWTH OF THE LANDSCHÜTZ ASCITES TUMOUR

W. GALBRAITH, E. MAYHEW AND E. M. F. ROE

*From the Chester Beatty Research Institute. Fulham Road. London, S.W.3*

Received for publication February 12, 1962

It has been shown (Roe, 1959) that Tragacanth Powder inhibits mouse ascites tumour growth *in vivo*. Samples from various species of *Astragalus* have been tested and growth inhibitory activity is present in all. It has been shown to be greater for the higher-grade commercial powders, but does not reside in various gums of different botanical origin which have been tested, nor in a variety of other polysaccharides. The inhibitory effect is destroyed by mild chemical and physical treatment of the powder and experiments to date suggest that it depends on the maintenance of a specific structure in the main macromolecular component of the gum, i.e., in one, or a mixture of polysaccharides. Details of the above experiments will be published separately. This paper is an account of further work concerning the mode of action of Tragacanth Powder (T.P.).

T.P. inhibits mouse ascites tumour growth when injected intraperitoneally. In the experiments reported below the Landschütz ascites tumour was used, inoculated in C+ (male) or C— (male or female) mice (C.B.R.I. strain).

The present report is divided into four sections :—

I. Dead cell counts.
II. Mitotic Index counts.
III. *In vitro–in vivo* experiments.
IV. Polysaccharide staining.

### *I. Dead cell counts*

Certain dyes are recognised as indicators of cell death. Lissamine Green has been the indicator preferred in these experiments as it is non-toxic in the concentrations employed (Goldacre and Sylvén, 1959; Holmberg, 1961).

Ascites tumour cell suspension from a single mouse was placed in both halves of a Burker counting chamber, diluted 1 : 2 in one side with an isotonic solution of the dye to act as a control, and in the other side with an isotonic solution of dye and T.P. in various concentrations. The percentages of cells stained after 5, 10, 20, 30, 40, 50 and 60 minutes could then be noted for both control and treated suspensions. Counts were made using T.P. concentrations of 0·1 and 1·0 mg. per ml. of cell suspension, dye concentrations of 1 : 200 and 1 : 2000, and tumours from 4 to 14 days old. A 4-day-old tumour is the youngest from which fluid may readily be extracted, and after about 14 days, untreated tumour-bearing mice usually die. The T.P. was added untreated, or deactivated by boiling for 5 minutes.

It was then thought desirable to repeat the dead cell counts after longer periods in contact with the T.P. than the 60 minutes previously attempted, and without the uncertainties (e.g. due to leucocytosis) of the *in vivo* experiments. For this purpose a culture apparatus was made, consisting of two identical culture vessels provided with a gas reservoir containing 5 per cent $CO_2$ in air, an air pump which gently agitated the fluid in the vessels to prevent the settlement of cells on the inner surfaces, and a U.V. lamp illuminating two quartz tubes in the gas pipes so that the culture vessels remained sterile and isolated from the non-sterile pump and gas reservoir. The cultures were maintained at 36°. Samples could be removed from the vessels by inserting a long syringe needle through a rubber teat without breaking the sterile conditions. It was possible, therefore, to remove and count a series of samples at intervals during an experiment and to note the change with time in the percentage of dead cells for control and treated samples, the T.P. being in the culture medium of one vessel. Earle's medium was used (Earle, 1943), i.e. a solution of inorganic salts and glucose only, as it was not necessary to maintain the culture for long periods or for the cells to multiply, and it was considered advisable to avoid the complications introduced by animal extracts. By these methods the cultures were easily maintained in a healthy state for over 24 hours, and occasionally up to 72 hours. Cell concentrations were 1·8–6·0 $\times$ $10^6$ per c.c. in these experiments and T.P. concentrations varied from 0·3 to 1·0 mg./ml.

However, in spite of the known inhibitory activity of T.P. *in vivo*, no significant differences could be found between the control and treated cells in any of the above experiments except possibly at 24 hours on occasions when the culture was moribund (Table I). Similar results were obtained in the few experiments in which Methylene Blue was used as an indicator of cell death.

Thus, since T.P. appears to have no toxic effect on the interphase tumour cells, it was decided to investigate possible effects on the Mitotic Index.

TABLE I.—*Percentage Deaths of Ascites Tumour Cells After Treatment* In Vitro *with T.P.* (*Indicator,* 1 : 2000 *Lissamine Green*)

| | Time in minutes | | | | | | |
|---|---|---|---|---|---|---|---|
| | 5 | 10 | 20 | 30 | 40 | 50 | 60 |
| Controls . . . . . | 1·1 | 1·8 | 2·2 | 2·4 | 2·5 | 2·7 | 2·9 |
| 0·1 mg./ml. T.P. . . . | 1·1 | 0·8 | 1·7 | 2·0 | 2·1 | 2·3 | 2·8 |
| 1·0 mg./ml. T.P. . . . | 1·7 | 2·0 | 2·3 | 2·4 | 2·5 | 2·7 | 2·8 |
| 1·0 mg./ml. T.P., de-activated | 1·0 | 1·7 | 2·1 | 2·2 | 2·2 | 2·4 | 2·7 |

| | Time in hours | | | | | | |
|---|---|---|---|---|---|---|---|
| | 0 | 1 | 2 | 3 | 4 | 6 | 24 |
| Controls . . . . . | 1·4 | 1·4 | 0·9 | 3·3 | 2·4 | 3·3 | 17·6 |
| 0·3 mg./ml. T.P. . . . | 1·6 | 1·0 | 0·8 | 1·7 | 2·7 | – | – |
| 0·5 mg./ml. T.P. . . . | 0·9 | 0·5 | – | 2·9 | – | 4·3 | 24·9 |
| 1·0 mg./ml. T.P. . . . | 1·3 | 0·8 | 0·6 | 2·0 | 1·8 | – | 22·9 |

*II Mitotic Index counts*

For this purpose aceto-orcein squashes were made at daily intervals from the ascites tumours of control mice and mice treated intraperitoneally with different doses of T.P. at the 7th day after tumour inoculation. Two factors confused the results : first, the great increase in neutrophils and lymphocytes in the treated mice and, to a lesser extent, in the controls as the tumour aged ; and secondly, the presence in the treated samples of ascites cells with pycnotic nuclei of two varieties, dense and lobed (d and l respectively in Fig. 1) which can be confused with the lymphocytes and neutrophils respectively. The appearance of the cytoplasm can be used to distinguish these, however, for the ascites cells are larger and have more granular cytoplasm than the while blood cells. Pycnosis is partly a matter of definition, since abnormal cells can be recognised which may not be considered fully pycnotic. A provisional definition was made whereby the absence of a recognisable nucleolus denoted a pycnotic nucleus.

The results are shown in Tables II (a) and (b) and Table III. From Table II (a) it can be seen that T.P. in the higher doses completely inhibits mitosis, while in lower doses there is moderate inhibition with recovery by the third day. Boiling the T.P. destroys the mitotic inhibitory activity. In one experiment, the stages of mitosis were also counted (Table II (b)). It can be seen that all stages, even prophase, are much less frequent in the treated cells. From Table III it is clear that the percentage of pycnotic nuclei increases with increasing doses of T.P. There is a large increase in the number of neutrophils and, to a lesser extent, in the lymphocytes also.

TABLE II (a).—*Mitotic Index Changes with Time for Different Doses of Tragacanth Powder per Mouse (Administered Intraperitoneally ; 7-day-old Tumour)*

| | | 24 hr. | 48 hr. | 72 hr. | 96 hr. |
|---|---|---|---|---|---|
| Mitotic index per cent | Controls | 1·1 | 1·5 | 1·1 | 1·2 |
| | 1 mg. | 0·2 | 0·2 | 0·8 | — |
| | 2 mg. | 0·3 | 0·2 | 1·1 | 1·3 |
| | 4 mg. | 0·0 | 0·0 | 0·0 | 0·0 |
| | 4 mg. de-activated | 0·8 | 1·2 | 1·5 | 1·1 |
| Thousands of Ascites cells counted | Controls | 34 | 30 | 18 | 10 |
| | 1 mg. | 20 | 20 | 8 | — |
| | 2 mg. | 14 | 10 | 4 | 4 |
| | 4 mg. | 8 | 10 | 6 | 2 |
| | 4 mg. de-activated | 8 | 8 | 8 | 8 |

TABLE II (b).—*Percentages of Ascites Tumour Cells in Various Mitotic Stages.*

| | | Prophase | Metaphase | Anaphase | Telophase | Cells counted |
|---|---|---|---|---|---|---|
| Controls, | 24 hr. | 0·33 | 0·51 | 0·14 | 0·09 | 6000 |
| | 48 hr. | 0·63 | 0·76 | 0·10 | 0·16 | 8000 |
| Treated, | 24 hr. | 0·09 | 0·03 | 0·00 | 0·00 | 8000 |
| (1 mg.) | 48 hr. | 0·00 | 0·00 | 0·02 | 0·00 | 8000 |

Fig. 1 shows the appearance of fixed and stained ascites tumour cell squashes from control and treated mice. Control cells show their normal appearance. Treated cells have numerous droplets in the cytoplasm and frequently pycnotic nuclei and in the later stages there is extensive cell breakdown.

### *III.* In vitro–in vivo *experiments*

It was of interest to discover whether, if T.P. was applied to the cells for a short time and then removed, its anti-tumour activity would be shown, and if so, what was the minimum period of application. Therefore, a series of experiments was performed in which ascites tumour cells were maintained for varying periods in the culture apparatus either with (B, Table IV) or without T.P. (A ; i.e. controls). The cultured cells were then removed, washed with saline, spun twice in

TABLE III.—*Changes with Time in the Percentages of Pycnotic Ascites Tumour Cells, Neutrophils and Lymphocytes for Different Doses of Tragacanth Powder (Conditions as in Table II)*

| | | 24 hr. | 48 hr. | 72 hr. | 96 hr. |
|---|---|---|---|---|---|
| Pycnotic ascites tumour cells | Controls | 1·1 | 1·1 | 1·2 | 0·6 |
| | 1 mg. | 3·1 | 6·6 | 5·2 | — |
| | 2 mg. | 3·1 | 3·6 | 5·3 | 10·5 |
| | 4 mg. | 13·7 | 5·0 | 13·4 | 18·9 |
| | 4 mg. de-activated | 0·3 | 0·4 | 0·3 | 0·4 |
| Neutrophils, per cent | Controls | 2·0 | 5·8 | 3·0 | 2·0 |
| | 1 mg. | 8·4 | 15·1 | 7·1 | — |
| | 2 mg. | 6·4 | 33·3 | 12·7 | 14·1 |
| | 4 mg. | 20·5 | 64·5 | 53·3 | 46·6 |
| | 4 mg. de-activated | 2·2 | 2·6 | 2·2 | 1·9 |
| Lymphocytes, per cent | Controls | 3·6 | 2·4 | 2·1 | 1·1 |
| | 1 mg. | 3·8 | 3·1 | 2·7 | — |
| | 2 mg. | 2·0 | 8·0 | 9·5 | 17·2 |
| | 4 mg. | 7·9 | 12·2 | 13·7 | 13·2 |
| | 4 mg. de-activated | 1·2 | 0·9 | 1·3 | 1·0 |
| Thousands of cells counted | Controls | 26 | 26 | 18 | 10 |
| | 1 mg. | 16 | 16 | 8 | — |
| | 2 mg. | 10 | 10 | 4 | 4 |
| | 4 mg. | 8 | 8 | 6 | 2 |
| | 4 mg. de-activated | 8 | 8 | 8 | 8 |

a centrifuge at 2650 g. for 3 minutes (to remove surplus T.P. from the treated cells, B) and resuspended in isotonic saline. The cells were inoculated into batches of fresh mice (A) and (B). A further batch of mice (C) was inoculated with cultured cells which had been treated with T.P., centrifuged as above and re-suspended in their treatment medium. Thus, in column A in Table IV are shown longevities of the mice inoculated with cells which had no contact with T.P. ; in column B are the corresponding figures for cells treated with T.P. for the length of time

TABLE IV.—*Survival of Mice in Days After Injection of T.P. Treated Ascites Tumour Cells*

| Length of *in vitro* treatment | *A* | *B* | *C* |
|---|---|---|---|
| 5 minutes | 24·0 | 19·2 | 41·4 |
| ½ hour | 15·4 | 24·4 | 33·2 |
| 5½ hours | 17·2 | 80·8+ | 90·0+ |
| 24 hours | 19·8 | 33·6 | 72·0+ |

### EXPLANATION OF PLATES.

FIG. 1.—Aceto-orcein squash preparations of ascites tumour cells.

A. Control.
B. 24 hours after treatment with Tragacanth Powder, 2 mg./mouse.
C. 48 hours after treatment.
D. 72 hours after treatment.

FIG. 2.—Ascites tumour cells stained with Hotchkiss' stain for polysaccharides.

A. Control.
B. Treated for 1 hour with 1 mg./ml. Tragacanth Powder. Centrifuged and washed before staining.
C. As B, but not centrifuged or washed before staining.

1

Galbraith, Mayhew and Roe.

2

Galbraith, Mayhew and Roe.

given in the table; and in column C are results for cells still in contact with T.P. while in the peritoneal cavity.

Each figure is the average survival in days of 5 mice. The figures with + signs indicate that not all the mice in those batches were dead when the experiment was terminated.

Control mice (A) inoculated with cells kept in saline for 5 minutes lived longer than the other A mice because it was not possible to use the culture apparatus for such a short experiment, and the tumour was therefore not so well protected from temperature shock and infection. Similarly, in the other three batches of A mice length of life increased with length of *in vitro* cell culturing due to progressive deterioration of the culture. Nevertheless, by comparing columns A and B, it can be seen that T.P. treatment of $\frac{1}{2}$ hour or more inhibited the tumour growth and increased the life of the mice, while 5 minutes' treatment had no effect. Column C mice showed greater longevity than B mice, since the treatment was continued intraperitoneally.

### *IV. Polysaccharide staining*

Since the main constituent of T.P. is a mixture of polysaccharides (James and Smith, 1945; Hirst, 1951), the Hotchkiss stain for polysaccharides (Glick, 1949) would be expected to give a positive reaction. Normal, untreated ascites tumour cells stained by Hotchkiss' method after Carnoy fixation show only slight positive staining, which appears in the cytoplasm, and, in some cells, is concentrated in spherical cytoplasmic granules.

To test for polysaccharide in T.P. treated tumour cells seven-day-old ascites tumour was removed from mice and incubated with T.P. solution, 1 mg./ml. in physiological saline at 37° C., for different times. The concentration of cells in the suspension was $2 \cdot 0 \times 10^7$ cells/c.c., and aliquots of the cell suspension were removed after incubation for 5 minutes, 30 minutes, 1 hour and 3 hours. Smears of these cells were then fixed in Carnoy, stained by the Hotchkiss method and examined under high power and compared with control tumour cells which had been incubated in saline alone. Further aliquots of the cell suspension incubated with T.P. were centrifuged, washed in saline to remove excess T.P. and fixed and stained as above.

Some results of these experiments are illustrated in Fig. 2. Adjacent to the cell membrane in cells incubated with T.P., positively staining material was found which was absent in the controls. The stained material seemed to form a layer on the cell membrane. In uncentrifuged cells the membrane appeared to be completely covered while in most of the washed cells the coating was reduced.

These results suggest that T.P. attaches itself to the cell membrane within the first half-hour of *in vitro* treatment. Even after centrifuging the staining remains visible showing that the binding is strong. Further experiments are in progress to detect any later penetration of the T.P. into the tumour cells, and any changes in their surface properties on treatment.

### DISCUSSION

From the above results it appears that the immediate action of T.P. on the ascites tumour cell is to attach itself to the cell membrane. The dead cell counts show that this does not alter the cell permeability to the vital stain Lissamine

Green ; and it is seen that control and treated cells " die ", i.e. become permeable to Lissamine Green, at the same rate. This indicates that the T.P. is not directly toxic to the cells. Belkin *et al.* (1959) obtained similar results when examining the damaging effects of various plant polysaccharides administered intraperitoneally to S37 mouse ascites tumours. Eosin was used as indicator in these experiments and treated and untreated tumours showed similar proportions of diffusely staining cells. Much more extensive vacuolisation of the cytoplasm was observed by these authors after treatment with the effective plant polysaccharides than in our experiments with T.P. It should be noted that T.P. (" commercial product ") was not effective in the experiments of Belkin *et al.* ; possibly the sample used was from a low-grade gum.

An increase in cell volume was also reported by these authors and this appears in our own preliminary experiments with medium T.P. doses (1 or 2 mg. in a 12-day-old tumour) although at higher doses (5 or 10 mg. in a 5-day-old tumour) cell shrinkage occurs. The changes in volume of the treated cells suggest an alteration in their permeability which is undetected by Lissamine Green tests and experiments are in progress to investigate the uptake of a different vital stain, i.e. the basic dye, acridine orange.

From the *in vitro–in vivo* experiments, it seems that T.P. acts on the cells within half an hour to reduce mitotic activity, while the histochemical staining shows that the polysaccharide component does not penetrate the cell in this time. Of course, non-staining constituents of the T.P. may penetrate the cell, or it may decompose slowly at the cell surface and its products may enter the cell. Alternatively, it is possible that T.P. constituents coating the cell prevent intake of essential nutrients or exit of toxic products of cell metabolism. Related experiments have been reported recently by Kornguth, Stahmann and Anderson (1961) using a fluorescent derivative of the basic polypeptide polylysine. This was incubated with Ehrlich ascites tumour cells, against which it shows a growth-inhibitory effect *in vivo*. Very little, if any, of this polypeptide appeared to enter the cells within the time of the experiment (10 minutes), but most of the material was observed bound to the cell surface in fluorescent clumps. It is possible that in experiments such as these the different polyelectrolytes are distinguishing different areas of specific charge on the cell membrane.

However it may occur, the net result of T.P. treatment of the ascites tumour cells is the suppression of mitosis, probably as a direct effect during interphase or early prophase which manifests itself in the prophase counts. Very few metaphase nuclei are found. The cells blocked during division degenerate into a pycnotic state and later disintegrate. Increase in white blood cell counts possibly occurs as a response to the breakdown products of the tumour cells, although Belkin *et al.* (1959) also noted a pronounced intraperitoneal leucocytosis after injection into the Sarcoma 37 ascites tumour of their T.P. sample, which did not cause cell swelling and vacuolisation.

### SUMMARY

Tragacanth Powder (T.P.) inhibits ascites tumour growth in mice. Evidence is put forward to show that T.P. becomes attached to the cell membrane, and that it acts as a mitotic block, probably indirectly, the direct effect occurring in the interphase or early prophase cell.

The authors are grateful to Professor A. Haddow, F.R.S., for his encouragement in this work.

This investigation has been supported by grants to the Chester Beatty Research Institute (Institute of Cancer Research : Royal Cancer Hospital) from the Medical Research Council, the British Empire Cancer Campaign, the Anna Fuller Fund, and the National Cancer Institute of the National Institutes of Health, U.S. Public Health Service.

### REFERENCES

BELKIN, M., HARDY, W. G., PERRAULT, A. AND SATO, H.—(1959) *Cancer Res.*, **19**, 1050.
EARLE, W. R.—(1943) *J. nat. Cancer Inst.*, **4**, 165.
GLICK, D.—(1949) ' Techniques of Histo- and Cyto-chemistry '. New York (Interscience Publishers), p. 44.
GOLDACRE, R. J. AND SYLVÉN, B.—(1959) *Nature, Lond.*, **184**, 63.
HIRST, E. L.—(1951) *Endeavour*, **10**, 106.
HOLMBERG, B.—(1961) *Exp. Cell Res.*, **22**, 406.
JAMES, S. P. AND SMITH, F.—(1945) *J. chem. Soc.*, 739, 746, 749.
KORNGUTH, S. E., STAHMANN, M. A. AND ANDERSON, J. W.—(1961) *Exp. Cell Res.*, 24, 484.
ROE, E. M. F.—(1959) *Nature, Lond.*, **184**, 1891.